# Identifying the Most Efficient Natural Fibre for Common Commercial Building Insulation Materials with an Integrated PSI, MEREC, LOPCOW and MCRAT Model

**DOI:** 10.3390/polym15061500

**Published:** 2023-03-17

**Authors:** Alptekin Ulutaş, Figen Balo, Ayşe Topal

**Affiliations:** 1 Department of International Trade and Business, Inonu University, 44210 Malatya, Turkey; 2 Department of METE, Engineering Faculty, Firat University, 23119 Elazıg, Turkey; 3 Department of Business, Nigde Omer Halisdemir University, 51240 Nigde, Turkey

**Keywords:** insulation material selection, LOPCOW, MCDM, MCRAT, MEREC, PSI

## Abstract

Building insulation is the most respected among the compatible and effective energy conservation technologies available today, as it also reduces yearly energy costs and negative environmental effects. A building envelope is made up of various insulation materials that are important in determining a building’s thermal performance. Proper insulation material selection concludes in less energy requisition for operation. The purpose of this research is to supply information about natural fibre insulating materials used in construction insulation to maintain energy efficiency, as well as to recommend the most efficient natural fibre insulation material. As in most decision-making problems, several criteria and alternatives are involved in insulation material selection, too. Therefore, we used a novel integrated multi criteria decision making (MCDM) model including the preference selection index (PSI), method based on the removal effects of criteria (MEREC), logarithmic percentage change-driven objective weighting (LOPCOW), and multiple criteria ranking by alternative trace (MCRAT) methods to deal with the complexity of numerous criteria and alternatives. The contribution of this study is that a new hybrid MCDM method is developed. Additionally, the number of studies using the MCRAT method is very limited in the literature; therefore, this study will provide more insights into and results of this method to the literature.

## 1. Introduction

Because of current global population growth trends, the building sector depletes an important amount of energy and resources. This case is expected to worsen in the future. This sector significantly contributes to ecological effects with greenhouse gas emissions, material transportation during structure events, the removal of generated solid waste from structure, and demolition events [1]. However, with increasing concerns about the scarcity of these sources and climate change, there is rising pressure on structure companies to decrease their environmental effect [2,3]. Buildings are built to serve specific functions; in some ways, they serve as intermediaries between our thermal environment and people [4]. A building’s thermal comfort is related to the materials used in its construction. It changes according to the effects of heat conduction, convection, and radiation. Building elements are greatly decorated, traditional structures and reproduce specific typologies [5]. Constructions can damage our health and environment by utilizing materials such as lead-based or toxicological paints and asbestos. As a result, ecologically beneficial building materials must be actively developed. Nowadays, green structures are increasing in demand to meet higher and more complex standards on energy efficiency, sustainability, and healthy comfort. To achieve this objective, an effective strategy is enhancing the insulation properties of the building envelope as well as using environmentally friendly materials in their lifetime [6]. The building envelope components serve as a heat barrier between the outdoors and indoors.

Building environmentally conscious and energy-efficient structures is a current trend in civil engineering. The attempts are made to preserve the environment’s natural patterns while using as many natural resources as possible in the construction process. Using new, innovative building materials that are environmentally friendly, have good end-use features, and are reasonably priced is one way to achieve these trends [7].

Modern insulation material requirements are primarily determined by the needs of people, building design features, being environmentally friendly, durability, safety, and functionality. Renewability serves as a fundamental resource and serves human continuity in architecture [8]. Building materials’ ecology poses a difficulty to both the efficacy of contemporary material use and the assessment of novel materials [9]. The novel combined science of ecological structure should aid in resolving the difficulties of forming and sustaining a superior quality building environment [10].

Insulation reduces heat exchange across any superficies, maintains the temperature desired, is too energy effective, and requires very little extra cooling and heating. Insulation also decreases noise conduction from area to area [11,12]. Only by using low-energy-intensive materials can energy consumption be reduced. Insulation materials have the potency to decrease energy consumption in the building sector. As a result, proper material selection and disposal is critical for the health, environmental, and social responsibility of a building. Environmental legislation linked to technological and user requisitions all over the world is increasing pressure on scientists and manufacturers to develop new environmentally friendly and maintainable materials to substitute present artificial fibres and reduce the reliance on fossil-sourced products. During the last years, the growing consciousness of maintainable and biodegradable materials, non-renewable resource scarcity, and foreseeable confidence in renewable sources has led to the focus on the improvement of environmental materials [13]. Overconsumption of petroleum-based materials contributes to the severe depletion of fossil resources [14,15]. To provide a comfortable room temperature with low carbon emissions, residential structures in developing nations must have enough thermal insulation. Therefore, choosing the right insulation material and treating it properly may make a significant impact, especially on the roof and wall structures that make up the building envelope [16]. Insulation from many natural thermal insulation materials is on par with or even more effective than insulation made from synthetic materials [17]. The specifiers are compelled to look into the effectiveness of natural insulating materials used for non-structural building elements due to availability and processing cost restrictions.

Environmentally friendly materials exhibit the advantages of less thermal conductivity and less density owing to their empty inner composition [18]. Further, the choice of a natural insulating material generally depends on technical factors such as thermal sensitivity, fire resistance, and carbon emission, but commercialisation and non-traditional insulation sources also play an important role in the development of efficient energy systems [19,20]. Natural fibre is a renewable resource, and the improvement of composites with natural fibre or environmentally friendly composite materials has recently become a hot issue owing to increased ecological concerns. Natural fibres are very capable materials that substitute artificial materials and their associated products for energy conservation and lighter weight implementations. Natural fibre materials have many specific properties and low prices, making them appealing for a variety of applications [21,22].

Natural fibre insulation can be utilised in construction similarly to other isolation in roof, wall, and floor build-ups. Fixing, cutting, and installation methods are also comparable. This provides installers transitioning from other insulation solutions with the ease of transfer and comparable installation timeframes. Natural fibre insulation is non-toxic and typically does not require protective clothes or respirators during installation, in contrast to oil- and glass-based insulation. This is especially useful for loft installations, where discomfort for installers is exacerbated by the necessity of full protection gear and frequent overheating. Moreover, natural fibre insulation materials are more durable when handled. Natural fibre-based insulation materials can be used as a healthier alternative for insulating contemporary building types such as passive houses, green buildings, and low-energy homes. Natural fibre insulation refers to a range of insulation materials made from natural materials such cellulose, wool, wood fibre, hemp, flax, and cotton. Since ancient times, materials made from natural resources, such as straw, reed grass, linen, hay, lichens, or hemp, have been utilised for thermal isolation. These insulation materials were frequently found on the construction exterior; lichens have been observed on several types of green roofs and facades. These were, however, supplanted by new synthetic materials, usually polymeric ones, such as inorganic, polyethylene, polyvinylchloride, and polystyrene synthetic materials such as mineral or glass wool, as civilisation advanced and the demand for well-made, high-quality buildings grew [23]. Products made of natural fibres can frequently be utilised in place of petrochemically or minerally sourced isolation. When utilised properly, organic fibre isolation products can provide acoustic and thermal isolation that is comparable to that provided by other isolation products, but with a smaller carbon footprint or even a negatory one and less health risks throughout the montage. They can also offer a vapour-permeable system and help with relative humidity control. When specified and installed properly, natural fibre insulation can involve a section of a vapour-permeable roof, wall, or floor mechanisms, providing a vapour-permeable construction layer. Unless completely covered through a damp proof membrane, organic insulations should not be utilised below the damp-proof course level. Organic fibres can help with efforts to lessen overheating in constructions since they have a good particular thermal storage ability. Using organic isolations’ good heat-mass in roofs can be especially important. Organic fibre insulations can also offer effective acoustic insulation due to their increased density and complex microstructure. Natural fibre insulations’ versatility is a key asset and ought to be taken into consideration when specifying or pricing products. Due to the natural materials’ low primary energy input values when compared to traditional fibres, they have very high potential from an ecological standpoint (for example glass fibres) [24,25]. These materials are a good substitute for the materials now in use, according to computer simulations. In certain studies, the cost of insulations was determined from an economic standpoint. The price of insulations with lower bulk densities was found to be totally similar to the price of generally available insulation on the market. Additionally, the latter enhance the thermo-insulating qualities of the insulation material during key times, which improves the overall thermal insulation qualities of the construction. Because of this, it can be claimed that these substances have both practical and financial benefits [26]. To provide the optimum value and benefits, these multi-functional qualities should be taken into consideration when selecting natural fibre isolation materials. The production of currently utilised thermo-insulating materials is highly expensive, energy-intensive, and resource-intensive. There are efforts being made to locate alternate material supplies, but local, naturally renewable materials appear to be the best option because they do not harm the environment and can be processed with low energy use. This also complies with the “20-20-20” climate and energy package adopted by the European Council in 2008 and added into law in 2009 [27]. Material sources’ sustainability makes readily renewable materials extremely important.

To take full advantage of these benefits, it is important to consider environmental friendliness, the use of readily renewable raw materials (or secondary raw materials), the ability to conserve general resources, and the ability to avoid non-renewable crude material usage. These materials aid in creating an environment that is healthy for human beings and are simply recyclable after serving their purpose in the structure [28].

Natural fibre insulation off-cuts can often be transported for bio-digestion or composting more, therefore leading to less landfill when used on-site because they do not require particular waste streams. Density is a high-quality indicator that is a problem to calculate, causing issues such as resistance to instalment, robustness in usage, capability to suit friction (materials are cut a little oversised to allow for overhead installation), thermal mass, and acoustic performance, even though some manufacturers do not frequently mention it. The extra heat storage capability of natural fibre insulation, which is twice as effective as that of mineral insulation in terms of weight for weight, may be useful in lightweight construction [29].

The embodied energy of insulation made of natural fibres is still unknown. Yet, because they are naturally occurring plant- or animal-based materials, their reuse and/or disposal has relatively little negative environmental impact [30].

It is commonly accepted that the thermal isolation qualities of organic fibre materials are solely humidity- and temperature-sensitive, and that higher temperatures and humidity reduce these qualities. The advantages and disadvantages of natural fibres are displayed in Table 1 [31].

Many papers are focalised around experimental research on natural fibre-based insulating materials that improve energy conservation and decrease greenhouse gas emissions to fulfil those standards [32].

Biomass use in the creation of thermos-isolation materials has been described in recent studies. Atif et al. (2019) created thermal lightweight insulations made of water-resistant hemp shiv, and the composite materials had the low thermal conductivity coefficients of 0.058–0.051 W/mK and reasonably high compressive strains ranging between 1.05 and 0.49 MPa [33]. The thermal conductivity value of the composite materials produced by Marie’s investigation of maize cob and hemp shiv residues as bio-sourced thermo-isolation materials varied between 0.1479 and 0.0675 W/mK [34].

Using a hot-pressing technique, Dang et al. created insulation fibreboards that are favourable to the environment and made of bone glue and bamboo fibres with protein as the main ingredient. The thermal conductivity value of the fibreboards was low, varying between 0.0582 and 0.0812 W/mK [35]. Muhammad et al. investigated a bio-composite made of a mix of maize stalk, fly ash, and magnesium phosphate cement. The findings showed that adding more corn stalks might increase the corn stalk composite materials’ thermal conductivity. When the corn stalks’ concentration reached 0.30, the thermal conductivity coefficients of two materials made from corn stalks were 0.0986 and 0.0510 W/mK, respectively [36].

By using hot press production procedures, pineapple leaf panels with organic rubber as a binder produced highly intriguing outputs, with thermal conductivity and density rising [37]. Durian insulation panels were obtained to have a mid-range thermal conductivity coefficient of 0.064 W/mK despite having a density of 428 kg/m^3^, but high-density insulation panels made by Muthuraj et al. from rice husks, wheat, wood fibres, combined with biodegradable multiple-composite resins, overall displayed rather good values.

The stiff panels by Bakatovich and Gaspar with medium density and highly competitive heat conductivity were produced from straw, sphagnum moss, and reeds and bound with NaSi [38].

Whilst other straw insulation panels have been published by Dissanayake et al. [39], these materials showed a somewhat higher thermal conductivity coefficient and density according to the heat capacity of the raw materials utilised. Straw bales indicated 0.067 W/mK for lower-density specimens. If verified, the created samples could, due to their expected high diffusivity, exist as greatly vying isolating materials in the evaluated area, including considerations of non-steady-state thermal behaviour.

Wei et al. looked at how a rice straw-based novel thermal insulation material’s qualities were impacted by board density, good heating, ambient temperature, and particle size. The outcomes displayed that boards with a density of 250 kg/m^3^ and a moisture amount of 14% had the most efficient mechanical and physical properties. The thermal performance of the thermal insulation boards was also good, with a thermal conductivity coefficient from 0.051 to 0.053 W/mK [40].

Two heat-isolation panels with lower density were created by Panyakaew and Fotios, one from bagasse and the other from coconut husk, both of which were constructed without the aid of chemical binders. According to the findings of their empirical study, the thermal conductivity coefficients of both insulation boards ranged between 0.068 and 0.046 W/mK, and at the lower end were comparable to those of more traditional insulating materials such as mineral-wool [41].

Cotton stalk fibres were used to make a binderless cotton stalk fibreboard (BCSF) by Zhou et al. using heat pressing rather than resins or other chemical additions. The boards’ thermal conductivity coefficients ranged between 0.0815 and 0.0585 W/mK, which are comparable to those of vermiculite and expanded perlite within the identical density interval. The panels were created at densities of 450–150 kg/m^3^ [42].

Andean feather grass was researched for Ichu’ thermal qualities as a regional and organic insulating material for rural homes by Charca et al. [43]. The findings showed that, for unidirectionally oriented fibres, the thermal conductivity ranged between 0.113 and 0.047 W/mK.

Experimental research was conducted by Briga-Sá et al. to determine whether fabric waste and this residue’s sub-waste, known as woven fabric sub-waste, might be used for heat isolation in building. The findings indicated that woven fabric waste had superior isolation qualities compared to woven fabric sub-waste, and that its thermal conductivity coefficient was comparable to that of more traditional heat isolation materials such as extruded-expanded polystyrene and mineral wool [44].

Processing methods do not affect natural fibre selection, but production can be completed with different operations. In the studies, a few methods that are mostly applied for the fabrication of natural fibres in excess by weight stand out. The qualities of the boards produced can be impacted by a few things throughout the binderless board manufacturing process. These variables include the duration, temperature, and pressing conditions in addition to the presence and particle sizes of other chemicals [45]. The procedure used to treat the fibres during the manufacturing process is referred to as treatment. Pre-treatment, on the other hand, describes a technique used to modify some of the qualities of fibres before their employment in the manufacturing process. In the creation of binderless boards, heat treatment is a typical production procedure. Nonetheless, extrusion and compaction procedures have been used to create boards without a binder. Better quality binderless boards are produced with the help of pre-treatment procedures such as steam explosion and a few others, especially from the viewpoint of dimensional steadiness. Pre-treatment techniques are also frequently employed as a substitute to grinding and pre-heating, which are both easier operations than vapour processing. According to earlier research about binderless panel manufacturing, hot-pressing is frequently used since it is less complicated than other techniques [46,47,48]. Heat implementation, which leads to the chemical elements of the crude materials employed, is the fundamental premise of hot-pressing. Essentially, the crude material is placed within a pattern, which is then set in a hydraulic hot-pressing machine. The material inside the pattern is then compressed in accordance with the pre-set parameters. Binderless boards with a compaction process can also be made using the extrusion method as well as with the steam and heat operations. Extrusion happens during the compression operation, in which the compression tool is heated and the loads for the process are put into it. The loads are then extruded through the die under a variety of circumstances [49]. Depending on factors such as the temperature of the pressing process and the chemical makeup of lignocellulose, the vapour pre-treatment operation has been shown to be beneficial for increasing wood-sourced composite materials’ dimensional steadiness [50,51,52]. Pre-treatment utilising grinding and pre-heating is another procedure to enhance binderless boards for lab-scale preparation. Microwave pre-heating of fibres prior to hot pressing is the easiest pre-treatment. The characteristics and inner-bond stress of fabricated panels are enhanced as a result.

Material selection has become a critical operation in engineering to obtain both customer satisfaction and successful maintainable planning [53,54,55]. Several constraints and factors limit the application of novel materials and bio-composite materials in a special industry [56,57,58,59,60]. As a result, selecting the most suitable material kind for private implementation is complicated work in which proper descriptions must be made utilising dual comparative studies, which is the decision-making foundation in various engineering sectors [61,62].

There are a few attributes for choosing component materials for a combined material, but the difficulty is in evaluating which ones to choose to acquire the best efficiency resolution from dedicated options [63]. Multi-criteria decision making (MCDM) methodologies can also be thought of when choosing composite materials’ elements because they suggest a logical proposal to authorised persons from a finite number of options. Numerous forms of MCDM modelling assess options’ efficiency and ensure the best prospective resolution amongst diverse options. Table 2 shows the MCDM studies of material selection decision problems in the literature.

In the contemporary world, a great number of diverse and various insulating materials are manufactured. Each material can be associated with one or the other group depending on the overall grouping attributes. Insulating materials’ composition, alike that of any matter, can be separated into organic, mineral, and composite (mineral and organic) groups. The selection of insulating materials is closely related to the technical, thermal, economic, and environmental parameters of insulation materials and so is significant in the lifecycle evaluation of a building.

In this study, the selection of natural fibres for building insulation implementations was conducted in a stepwise strategy. Before starting the analysis, information about the comprehensive features of natural fibres was summarised. Nine criteria, which are the vapour diffusion resistance factor (VDRF), sound absorption coefficient (SAC), embodied carbon (EC), embodied energy (EE), cost (C), reaction to fire (RF), specific heat capacity (SHC), thermal conductivity (TC), and density (D) were noted for the assessment of the most effective natural fibre. After that, an integrated MCDM analysis, including the preference selection index (PSI), method based on the removal effects of criteria (MEREC), logarithmic percentage change-driven objective weighting (LOPCOW) and multiple criteria ranking by alternative trace (MCRAT), was used to anticipate the weights of the criteria relying on the notional significance of one attribute over another and the rank of alternatives.

## 2. Materials and Methods

A material with thermal insulation has important features, explained below, that provide satisfying efficiency throughout a building’s life: The material should be appropriate for continual use at maximal operational temperatures without the deterioration of its features physically; it must be well-anchored, void-free, and tight. Replacement and removal should be required only during plant maintenance or alteration; it should have enough effect resistance and flexural strength to allow for safe implementation and transportation. The material should be non-flammable; it should not create health hazards or discomfort to running staff; it should have enough compressed strength to resist regional loads such as ladders, foot traffic, and so on; it should not be abrasive to pipe work and plants if wet through water/steam or rain leakage; it should not be continually harmed if polluted by water and humidity [80].

The following parameters were used as criteria in the selection of the most effective natural fibre in terms of thermal insulation, considering the properties outlined above and which should be in the thermal insulation material [81,82,83,84].

A material’s VDRF is a gauge of its unwillingness to allow water vapour to pass through. Because vapour strength is affected by material thickness, it can solely be excerpted for a material’s specific thickness.

SAC is utilised to assess a material’s capability to absorb sound. It is the adsorbed energy’s rate of incident energy. Provided that acoustic energy can be completely absorbed, it is equal to one.

The lifecycle of energy-sourced pollutants (such as carbon-dioxide), which are a consideration within the framework of climate change and global warming, can be examined. As a consequence, the concept of EC emerges [85]. Carbon dioxide gasses correlate with construction processes and materials over the course of a building’s lifecycle or substructure and are called EC.

EE is the total energy used to create a material or product, including mining, manufacturing, and transportation. The total energy needed to produce a finished product contains the energy used to extract, grow, transport, and manufacture it. To achieve a truly low-energy home, embodied energy must be considered when selecting materials and construction systems. Pollutant emissions and energy may be noted as embodied within materials. Therefore, embodied energy can be defined as the required amount of energy to provide the material.

C efficacy through utilising renewable and recycled material can provide the industry and community with advantages such as diminishing the air pollution level and creating less health problems.

RF displays whether or not a material provides fuel to the fire prior to the flash-over. This is divided into 7 classes, numbered F to A_1_, with F containing promptly combustible materials and A1 containing non-flammable materials (Euroclass; F/E/D/C/B/A_2_/A_1_). The rating of the fire resistance of insulation materials in this research is given in Table 3.

The required amount of heat to raise the temperature value per unit mass is called SHC. Characteristically, it is the heat amount in Joules required to increase one gram of the sample’s temperature by one degree Celsius or Kelvin.

TC is identified as the amount of heat that fluxes per unit time.

D is the material mass per unit volume. When thermal conductivity and density are decreased, thermal insulation improves. Table 4 shows the criteria and alternatives used in this study.

### 2.1. PSI Method

The application of PSI method is explained below [140].

Step 1-1: Decision matrix (B) is formed. Equation (1) presents matrix.

(1) $$B={\left[b_{ij}\right]}_{m\times n}$$

Step 1-2: Normalisation of decision matrix is done with Equation (2) (beneficial criteria) and (3) (non-beneficial criteria).

(2) $$b_{ij}^{*}=\frac{b_{ij}}{max\left(b_{ij}\right)}$$

(3) $$b_{ij}^{*}=\frac{min\left(b_{ij}\right)}{b_{ij}}$$

Step 1-3: Mean values of normalised values are computed by Equation (4).

(4) $${\bar{b}}_{j}^{*}=\frac{\sum_{i=1}^{m}b_{ij}^{*}}{m}$$

Step 1-4: Preference variation value ($\rho_{j}$) for each insulation material is calculated.

(5) $$\rho_{j}=\overset{m}{\underset{i=1}{\sum }}{\left(b_{ij}^{*}-{\bar{b}}_{j}^{*}\right)}^{2}$$

Step 1-5: Equation (6) is used to calculate the deviation ($\delta_{j}$) in preference value for each criterion.

(6) $$\delta_{j}=\left|1-p_{j}\right|$$

Step 1-6: Criteria weights ($w_{jPSI})$ are calculated with Equation (7).

(7) $$w_{jPSI}=\frac{\delta_{j}}{\sum_{j=1}^{n}\delta_{j}}$$

### 2.2. MEREC Method

The steps below present MEREC method [141].

Step 2-1: Decision matrix (B) is created with Equation (1).

Step 2-2: Decision matrix is normalised by Equations (3) (beneficial criteria) and (2) (non-beneficial criteria).

Step 2-3: The alternatives’ total performance ($k_{i}$) is calculated.

(8) $$k_{i}=\ln\left(1+\left(\frac{1}{m}\sum_{j}\left|\ln\left(b_{ij}^{*}\right)\right|\right)\right)$$

Step 2-4: By eliminating each criterion, the performances of alternatives ($k_{ij}^{*}$) are calculated.

(9) $$k_{ij}^{*}=\ln\left(1+\left(\frac{1}{m}\sum_{k,\;k\neq 1}\left|\ln\left(b_{ij}^{*}\right)\right|\right)\right)$$

Step 2-5: Equation (10) shows how the total absolute deviations $(A_{j})\;$are determined.

(10) $$A_{j}=\sum_{i}\left|k_{ij}^{*}-k_{i}\right|$$

Step 2-6: Equation (11) is used to determine the weights of the criteria ($w_{jMEREC})$.

(11) $$w_{jMEREC}=\frac{A_{j}}{\sum_{K}A_{k}}$$

### 2.3. LOPCOW Method

LOPCOW method has been applied with the steps below [142].

Step 3-1: Decision matrix (B) is created with Equation (1).

Step 3-2: Decision matrix is normalised by Equations (12) (beneficial criteria) and (13) (non-beneficial criteria).

(12) $$c_{ij}=\frac{b_{ij}-b_{min}}{b_{max}-b_{min}}$$

(13) $$c_{ij}=\frac{b_{max}-b_{ij}}{b_{max}-b_{min}}$$

Step 3-3: Each criterion’s percentage values ($p_{ij}$) are determined by Equation (14).

(14) $$p_{ij}=\left|\ln\left(\frac{\sqrt{\frac{\sum_{i=1}^{m}c_{ij}^{2}}{m}}}{\sigma }\right).100\right|,$$

where the standard deviation and number of alternatives are denoted by σ and m, respectively.

Step 3-4: Weights of criteria ($w_{jLOPCOW})$ are found with Equation (15).

(15) $$w_{jLOPCOW}=\frac{p_{ij}}{\sum_{i=1}^{n}p_{ij}}$$

The following equation will be used to combine the three criteria weights (PSI, MEREC, and LOPCOW) [143]. $w_{jcomb}$ represents the combined weights of the criteria in Equation (16).

(16) $$w_{jcomb}=\frac{w_{jPSI}w_{jMEREC}w_{jLOPCOW}}{\sum_{j=1}^{n}w_{jPSI}w_{jMEREC}w_{jLOPCOW}}$$

### 2.4. MCRAT Method

The steps below present MCRAT method [144].

Step 4-1: Decision matrix (B) is created with Equation (1).

Step 4-2: Decision matrix is normalised by Equations (2) (beneficial criteria) and (3) (non-beneficial criteria).

Step 4-3: The weighted normalisation values ($v_{ij}$) are computed with Equation (17), and Equation (18) shows weighted normalised matrix (D).

(17) $$v_{ij}=w_{jcomb}\times b_{j}^{*}$$

(18) $$V={\left[v_{ij}\right]}_{m\times n}$$

Step 4-4: The ideal alternative is determined with Equation (19).

(19) $$y_{j}=max\left(\left.v_{ij}\right|1\leq j\leq n\right)$$

The set of ideal alternatives is shown as follows:

(20) $$Y=\left\{y_{1},\;y_{2},\ldots ,\;y_{j}\right\}$$

Step 4-5: This step entails breaking down the ideal alternative into two subgroups with Equation (21), and Equation (22) represents the ideal alternative.

(21) $$Y=Y^{max}\cup Y^{min}$$

(22) $$Y=\left\{y_{1},\;y_{2},\ldots ,\;y_{k}\right\}\cup \left\{y_{1},\;y_{2},\ldots ,\;y_{h}\right\};k+h=j,$$

where k shows the number of criteria and h=n−k.

Step 4-6: This step entails breaking down of other alternatives with Equations (23) and (24).

(23) $$K=K_{İ}^{max}\cup K_{İ}^{min}$$

(24) $$K=\left\{k_{1},\;k_{2},\ldots ,\;k_{ik}\right\}\cup \left\{k_{1},\;k_{2},\ldots ,\;k_{ih}\right\}$$

Step 4-7: Equations (25) and (26) are used to determine each component of the ideal alternative’s magnitude.

(25) $$Y_{k}=\sqrt{y_{1}^{2}+y_{2}^{2}+\ldots y_{k}^{2}}$$

(26) $$Y_{h}=\sqrt{y_{1}^{2}+y_{2}^{2}+\ldots y_{h}^{2}}$$

The magnitude of each alternative is also calculated with the same approach using Equations (27) and (28).

(27) $$E_{k}=\sqrt{e_{i1}^{2}+e_{i2}^{2}+\ldots e_{ik}^{2}}$$

(28) $$E_{h}=\sqrt{e_{i1}^{2}+e_{i2}^{2}+\ldots e_{ih}^{2}}$$

Step 4-8: Matrix T representing the best possible alternative components is developed with Equation (29).

(29) $$T=\left[\begin{array}{cc} Y_{k} & 0\\ 0 & Y_{h} \end{array}\right]$$

Step 4-9: Matrix $S_{i}\;$representing the component of alternative is developed with Equation (30).

(30) $$S_{i}=\left[\begin{array}{cc} E_{ik} & 0\\ 0 & E_{ih} \end{array}\right]$$

Step 4-10: Matrix $Z_{i}\;$is developed with Equation (31).

(31) $$Z_{i}=T\times S_{i}=\left[\begin{array}{cc} z_{11;i} & 0\\ 0 & z_{22;i} \end{array}\right]$$

Step 4-11: The matrix $Z_{i}$′s trace is calculated with Equation (32).

(32) $$tr\left(Z_{i}\right)=z_{11;i}+z_{22;i}$$

## 3. Results

In this study, we applied an integrated MCDM model consisting of the PSI, MEREC, LOPCOW and MCRAT methods. The insulation materials analysed in this study are commonly used in commercial buildings. VDRF, SAC, EC, EE, C, RF, SHC, TC, and D are the criteria used in this study to assess 20 insulation materials. Only the first two criteria (VDRF and SAC) are beneficial criteria; the rest of them are non-beneficial. The decision matrix is shown in Table 5. The values in the decision matrix shown in Table 5 are derived from Table 4. The values of the Aerogel material in the EC and EE criteria are interval values. The arithmetic mean of these values was used. Other values in the decision matrix were taken directly from Table 4.

In this study, combined weights were used in analysis. First, the PSI, MEREC, and LOPCOW methods were applied to the decision matrix shown in Table 5 to calculate the weights of each criterion. Then, the weights obtained from the three methods were combined with Equation (16). The weights from the three methods and the combined weights are presented in Table 6.

After the weights of the criteria were obtained, the MCRAT method was used to rank insulation the materials. The matrix was normalised by applying Equations (2) and (3) to Table 5. Table 7 presents the normalised matrix.

With Equation (17), the weighted normalised matrix shown in Table 8 was created.

The ideal alternative was identified with Equation (19), and the results are presented in Table 9.

Table 10 presents the decomposition of the ideal alternatives.

With Equations (23) and (24), the alternatives were decomposed. Then, the following stage in this selection procedure was to use Equations (25) and (26) to determine the magnitude of the ideal alternative components. Table 11 shows the magnitude levels of the ideal alternatives.

$E_{k}$ and $E_{h}$ given in Table 12 represent the magnitude of each alternative. They were computed using Equations (27) and (28). Equations (29) and (30) were used to calculate $T_{k}$ and $T_{h}$. Finally, the values for trace of matrix $Z_{i}$ were found with Equation (32), and the alternatives were ranked accordingly. The results of the MCRAT method are indicated in Table 12.

## 4. Comparative Analysis

A new integrated MCDM model including the PSI, MEREC, LOPCOW, and MCRAT methods has been developed, examined, and verified in this study using an insulation material selection problem. While PSI, MEREC, and LOPCOW were used to determine the combined weights, MCRAT was used to rank the alternatives. By using rank correlation, the findings of MCRAT were compared to those from the three popular and well-known MCDM methods: MARCOS, ARAS, and CRADIS. Table 13 shows the comparative analysis.

According to the results of comparative analysis, the MCRAT technique has high correlations with MARCOS, ARAS, and CRADIS, which are 0.970, 0.951, and 0.966, respectively. Sheep wool was identified as the first-ranking alternative among all the techniques examined. The same holds true for wood fibre, which was ranked as the second alternative among all the techniques and for hemp, which was ranked as the third alternative. The average order of the alternatives is maintained throughout the findings of all the techniques since there are no significant differences in ranking orders.

## 5. Sensitivity Analysis

Following the process outlined by Görçün et al., we first adjusted the weights of each criterion to test the stability of the suggested model [145]. To do this, the criteria weight coefficient was adjusted by 10% in each scenario, starting with the most significant criterion, until the criterion’s weight was equal to 0. Each criterion had 10 scenarios. The remaining criteria were then added to the difference value obtained after decreasing the criterion weight to ensure that the sum of the criteria would equal one. This method considers any adjustments to the relative importance of each criterion. The following basic steps of the proposed framework are illustrated in Equations (33)–(35) [145]:

(33) $$w_{fv}^{1}=w_{pv}^{1}-\left(w_{pv}^{1}.m_{v}\right)$$

(34) $$w_{nv}^{2}=\frac{\left(1-w_{fv}^{1}\right)}{n-1}+w_{pv}^{2}$$

(35) $$w_{fv}^{1}+\sum w_{nv}^{2}=1$$

**Figure 1:**
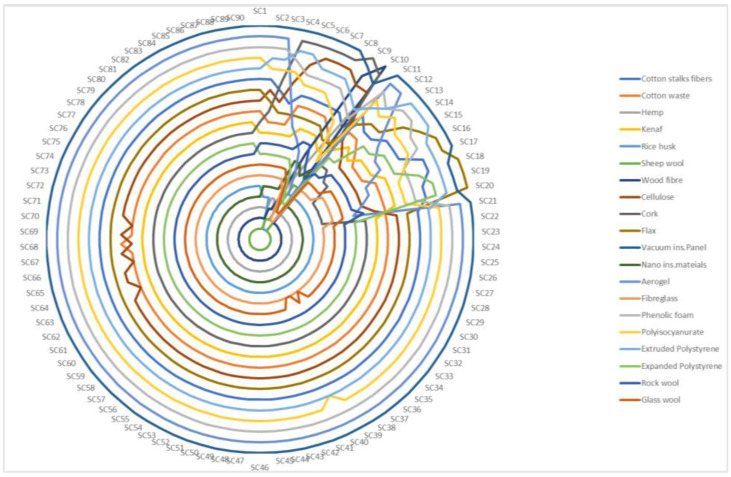
The ranking of alternatives by taking different criteria weights into consideration.

In Figure 1, changes in the overall ranking performance of the alternatives were shown when the proposed frame was used. The rankings of all the insulating materials changed at least once. This shows that the change in the weights of the criteria caused changes in the ranking of the insulation materials. As a result, it has been revealed that the MCRAT method is sensitive to changes in criteria weights. As an example, the insulation materials in the first two rows are as follows: the sheep wool insulation material ranked first in the SC1-SC4 and SC11-SC90 scenarios. The rice husk insulation material ranked first in the SC5-SC10 scenarios. Likewise, the wood fibre insulation material took the second place in the SC1-SC4 and SC11-SC90 scenarios, the sheep wool insulation material took the second place in the SC5-SC7 scenarios, and the fibreglass insulation material took the second place in the SC8-SC10 scenarios. Each line in Figure 1 represents a ranking. The innermost line indicates the 1st rank, and the outermost line indicates the 20th rank. It should be noted that the light blue-coloured line of the rice husk material was the first line in the SC5-SC10 scenarios. In other words, this material took the first rank. The outermost line, in other words, the line showing the last rank varied with respect to the change in criteria weights. SC1-SC8, SC11-SC17, and SC21-SC90 have a dark blue-coloured line with the vacuum-insulated panel material as the last line. In SC9, the dark grey-coloured line of the cork material is placed as the last line, while in SC10, the very dark blue-coloured line of the wood Fibre material is the last line. In SC18-SC20, the dark brown-coloured line of the flax material is the last line. In other words, the mentioned materials took the last place; that is, they took the 20th place in the mentioned scenarios.

## 6. Conclusions

Green manufacturing practices with correct natural fibre insulation material selection can protect future generations, providing them with environmental protection. The operation of material selection is dependent on the decision-making and application tools used, which may affect the outcome. In this study, we developed a new integrated MCDM model including the PSI, MEREC, LOPCOW, and MCRAT methods to select an ideal insulation material alternative. There were nine main selection criteria utilised to decide the best natural insulation material out of 20 alternatives. This study has two contributions. The novel MCDM technique was created in this study. Additionally, the challenge of choosing an insulation material was first solved by combining the PSI, MEREC, LOPCOW and MCRAT methods.

The results indicated that sheep wool was the best alternative in the selection process, followed by wood fibre and hemp. The rest of the alternatives were sorted as nano insulation materials, rice husk, fibreglass, glass wool, rock wool, expanded polystyrene, cork, kenaf, cotton waste, cellulose, flax, cotton stalks fibres, extruded polystyrene, polyisocyanurate, phenolic foam, aerogel, and vacuum-insulated panel. It was found in the sensitivity analysis that VDRF and SAC are important criteria, and changes to their weights may have an impact on the rankings. When we changed the weights of VDRF and SAC, the ranking of most alternatives changed. When EC weights were adjusted, only the rank of extruded polystyrene and glass wool were somewhat altered. Additionally, the weight alterations in SHC caused a shift in the ranking of cellulose.

The MARCOS, ARAS, and CRADIS techniques were compared with the developed model. The comparisons have the following correlation coefficients: MCRAT–MARCOS (0.970), MCRAT–ARAS (0.951), and MCRAT–CRADIS (0.966). The developed model findings and those from other MCDM techniques exhibit a significant correlation. Consequently, it was found that the suggested model produced reliable findings.

The advantages of the MCDM model proposed in this study are as follows. The proposed MCDM model is a robust model. By means of this model, detailed and reliable results can be obtained. The process of each method used in the model is easy. The disadvantages of the MCDM model proposed in the study are as follows: (1) An analysis of dependent variables is not possible. (2) Computation using the MCRAT method takes a long time. (3) Qualitative values must be converted to quantitative values for computing.

More than one objective weighting method (PSI, MEREC, and LOPCOW) was used in this study. It is thought that more reliable results were obtained, unlike when using a single objective weighting method. Namely, in the single objective weighting method, only the results found by that method would be trusted and the processes would continue with the results of this weighting method. However, in this study, instead of the results of a single objective weighting method, the results of three objective weighting methods were included in the process. Thus, it is thought that more reliable and rigorous results were obtained in this study.

This study supplies an analytic framework for efficiently assisting designers or material engineers in selecting the best natural fibre among commercially available alternatives to choose new materials for the building envelope insulation material process. Other details could be added to acquire a more comprehensive list of selection criteria and thus more comprehensive outputs to research for future improvement. Additionally, other MCDM-based techniques may be used to address insulation material selection issues in future studies. In addition, in this study, we have used objective data; in future studies, subjective data and a combination of both can be considered. Further, the developed MCDM model (PSI-MEREC-LOPCOW-MCRAT) is reliant on the use of crisp numbers, which is one of the constraints of the proposed model.

Although a detailed hybrid MCDM model was used in this study, only insulation materials were evaluated in this study. Future studies can use the proposed MCDM model for different performance measurement problems. In addition, unfortunately, experimental results were not included in this study. Future studies can analyse experimental results with the proposed hybrid MCDM model.

## Figures and Tables

**Table 1:** The advantages and disadvantages of natural fibres.

Advantages	Disadvantages
They absorb moisture and water, which makes them easily at risk of damage.	They are not very durable.
They have an incompatible nature.	Natural fibres’ strength is very low.
They have low density and are lightweight.	They can be harmed by insects and moths.
The are renewable, biodegradable, and eco-friendly.	They do not have wrinkle-free properties.
They are abundant in nature.	They are affordable, but more expensive than artificial fibres.
They have insulation and thermal features.	
They do not create allergic reactions or irritate the skin.	
The production process emits less CO_2_ emissions.	
The production process requires less energy.	
They have electrical resistance features.	
They do not release poisonous gases when burnt.	

**Table 2:** Some MCDM research on material selection.

Area	Aim	Method	Reference
Construction	To assess the criteria weights for choosing a combined material.	AHP	[64]
Meal Packaging	To choose the most proper plant fibre amongst nine natural fibres.	AHP	[65]
Automotive Anti-Roll Bars	To select thermo-polymers for natural fibre-supported plastic composite materials.	AHP	[66]
Construction	To research flax fibre elements with the best efficiency resolution in combined materials.	TOPSIS	[67]
Construction	To compare features of diverse insulation materials focusing on operational energy, lifecycle cost, carbon, embodied energy, and an optimal insulation material that is comfortable to choose.	System optimization	[68]
Maintainable Insulations	To assess the effect of maintainable insulations on financial and ecological appropriateness.	Criteria classes	[69]
Construction	To address the maintainability, researchers displayed the material source evaluation and determiners’ requirement, i.e., parameters, inputs, efficiency determiners of criterions in material selection.	Criteria classes	[70]
Construction	To assess six insulating material options for buildings’ design resolutions in Lithuania.	TODIM-SWARA	[71]
Construction	To identify insulating materials’ orientation for maintainability in constructions.	Lifecycle analysis	[72]
Food Packaging	To identify the best natural fibre strengthening material, the mechanism for natural fibre material selection in bio-plastic composite materials strengthening.	AHP	[73]
Historical Constructions	The arrangement of five contemporary insulating materials for strengthening historical constructions. Eco-wool was sorted as the most efficient insulating material resolution.	TOPSIS-Grey methods	[74]
Construction	The accountancy of ecological issue tools for lifecycle databases and energy evaluation to promote consciousness amongst consumers in providing energy performance.	Debated diverse methods	[75]
Construction	The most desirable alternatives displayed are styrofoam, glass wool and woodwool.	VIKOR	[76]
Construction	The prediction of the effects on ecology owing to embodied carbon which gives rise to power usage.	Criteria classes	[77]
Insulating Materials	To select an environmentally friendly insulation material.	AHP–TOPSIS	[78]
Construction	To define the most effective resolution for flax fibre elements.	TOPSIS	[79]

**Table 3:** The rating of fire resistance of insulation materials in this research [86].

Euroclass	Contribution to Fire	Degree
A1	Non-combustible	1
A2	Limitedly combustible—no flashover	2
B	No flashover	3
C	Flashover after 10 min	4
D	Flashover before 10 min	5
E	Flashover before 2 min	6
F	No performance determined	7

**Table 4:** Insulation materials commonly used in commercial buildings [19].

Insulation Type	VDRF	SAC	EC	EE	C	RF	SHC	TC	D	Reference
	-	-	kgCO_2_ e⋅kg^−1^	MJ/kg	$/m^3^	-	J/goC	W/m K	kg/m^3^	-
Cotton stalk fibres	1.2	0.37	2.55	46	18.8	6	0.13	0.070	300	[87,88]
Cotton waste	1.5	0.81	1.28	27.1	19.3	6	1.6	0.041	32	[89,90,91]
Hemp	5.5	0.56	0.14	18.71	17.2	6	1.75	0.081	63	[92,93,94]
Kenaf	1.8	0.63	1.34	30.88	17	6	0.95	0.035	105	[95,96,97]
Rice husk	2	0.41	0.6	1.36	5	1	1.95	0.064	150	[98,99,100,101]
Sheep wool	4.5	0.59	0.12	5.4	24	6	1.5	0.046	15	[102,103,104,105,106]
Wood fibre	3.0	0.21	0.124	20.3	32.2	6	2	0.044	160	[107,108]
Cellulose	2.35	0.72	1.07	6.9	24.6	4	1.45	0.039	55	[109,110,111]
Cork	17.5	0.62	0.82	26	35.2	6	1.6	0.040	110	[112,113,114]
Flax	3.14	0.69	20	39.5	16.5	4	1.6	0.061	60	[115,116,117,118]
Vacuum-insulated panel	340	0.2	8.65	187.5	131	2	0.8	0.057	195	[119,120]
Nano insulation materials	5	0.5	0.22	2.04	3000	4	1.0	0.027	230	[121,122,123]
Aerogel	3.75	0.66	4-3	5.3-9	137.5	2	0.1	0.017	110	[124,125,126,127,128,129]
Fibreglass	1.15	1.25	1.24	22.4	12	1	0.9	0.040	55	[130,131,132,133]
Phenolic foam	35	0.4	5.68	86	23	4	1.35	0.021	100	[134,135,136]
Polyisocyanurate	103	0.1	5.5	69.8	22	3	1.45	0.023	38	[103,137]
Extruded polystyrene	125	0.43	7.55	89	20.5	6	1.58	0.035	36	[98,103,138]
Expanded polystyrene	60	1.93	6.8	103.9	12.8	6	1.25	0.035	34	[98,103,138,139]
Rock wool	1.15	0.59	1.05	16.8	16	2	0.9	0.037	120	[98,103,138]
Glass wool	1.14	0.62	1.24	22.4	12	1	0.9	0.040	55	[103,140]

**Table 5:** Decision matrix.

Insulation Type	VDRF	SAC	EC	EE	C	RF	SHC	TC	D
Cotton stalk fibres	1.2	0.47	2.55	46	18.8	6	0.13	0.07	300
Cotton waste	1.5	0.91	1.28	27.1	19.3	6	1.6	0.041	32
Hemp	5.5	0.66	0.14	18.71	17.2	6	1.75	0.081	63
Kenaf	1.8	0.73	1.34	30.88	17	6	0.95	0.035	105
Rice husk	2	0.51	0.6	1.36	5	1	1.95	0.064	150
Sheep wool	4.5	0.69	0.12	5.4	24	6	1.5	0.046	15
Wood fibre	3	0.31	0.124	20.3	32.2	6	2	0.044	160
Cellulose	2.35	0.82	1.07	6.9	24.6	4	1.45	0.039	55
Cork	17.5	0.72	0.82	26	35.2	6	1.6	0.04	110
Flax	3.14	0.79	20	39.5	16.5	4	1.6	0.061	60
Vacuum-insulated panel	340	0.3	8.65	187.5	131	2	0.8	0.057	195
Nano insulation materials	5	0.6	0.22	2.04	3000	4	1	0.027	230
Aerogel	3.75	0.76	3.50	7.15	137.5	2	0.1	0.017	110
Fibreglass	1.15	1.35	1.24	22.4	12	1	0.9	0.04	55
Phenolic foam	35	0.5	5.68	86	23	4	1.35	0.021	100
Polyisocyanurate	103	0.1	5.5	69.8	22	3	1.45	0.023	38
Extruded polystyrene	125	0.53	7.55	89	20.5	6	1.58	0.035	36
Expanded polystyrene	60	2.03	6.8	103.9	12.8	6	1.25	0.035	34
Rock wool	1.15	0.69	1.05	16.8	16	2	0.9	0.037	120
Glass wool	1.14	0.72	1.24	22.4	12	1	0.9	0.04	55

**Table 6:** Criteria weights.

Weights	VDRF	SAC	EC	EE	C	RF	SHC	TC	D
$w_{jPSI}$	0.0166	0.0832	0.3656	0.0634	0.0614	0.2265	0.0685	0.0847	0.0301
$w_{jMEREC}$	0.1099	0.1086	0.1601	0.1276	0.3002	0.0317	0.0407	0.042	0.0791
$w_{jLOPCOW}$	0.0204	0.078	0.1598	0.149	0.2097	0.0546	0.0756	0.1218	0.1311
$w_{jcomb}$	0.0023	0.0427	0.5664	0.073	0.2341	0.0237	0.0128	0.0262	0.0189

**Table 7:** The normalised matrix.

Insulation Type	VDRF	SAC	EC	EE	C	RF	SHC	TC	D
Cotton stalk fibres	0.0035	0.2315	0.0471	0.0296	0.266	0.1667	0.7692	0.2429	0.05
Cotton waste	0.0044	0.4483	0.0938	0.0502	0.2591	0.1667	0.0625	0.4146	0.4688
Hemp	0.0162	0.3251	0.8571	0.0727	0.2907	0.1667	0.0571	0.2099	0.2381
Kenaf	0.0053	0.3596	0.0896	0.044	0.2941	0.1667	0.1053	0.4857	0.1429
Rice husk	0.0059	0.2512	0.2	1	1	1	0.0513	0.2656	0.1
Sheep wool	0.0132	0.3399	1	0.2519	0.2083	0.1667	0.0667	0.3696	1
Wood fibre	0.0088	0.1527	0.9677	0.067	0.1553	0.1667	0.05	0.3864	0.0938
Cellulose	0.0069	0.4039	0.1121	0.1971	0.2033	0.25	0.069	0.4359	0.2727
Cork	0.0515	0.3547	0.1463	0.0523	0.142	0.1667	0.0625	0.425	0.1364
Flax	0.0092	0.3892	0.006	0.0344	0.303	0.25	0.0625	0.2787	0.25
Vacuum-insulated panel	1	0.1478	0.0139	0.0073	0.0382	0.5	0.125	0.2982	0.0769
Nano insulation materials	0.0147	0.2956	0.5455	0.6667	0.0017	0.25	0.1	0.6296	0.0652
Aerogel	0.011	0.3744	0.0343	0.1902	0.0364	0.5	1	1	0.1364
Fibreglass	0.0034	0.665	0.0968	0.0607	0.4167	1	0.1111	0.425	0.2727
Phenolic foam	0.1029	0.2463	0.0211	0.0158	0.2174	0.25	0.0741	0.8095	0.15
Polyisocyanurate	0.3029	0.0493	0.0218	0.0195	0.2273	0.3333	0.069	0.7391	0.3947
Extruded polystyrene	0.3676	0.2611	0.0159	0.0153	0.2439	0.1667	0.0633	0.4857	0.4167
Expanded polystyrene	0.1765	1	0.0176	0.0131	0.3906	0.1667	0.08	0.4857	0.4412
Rock wool	0.0034	0.3399	0.1143	0.081	0.3125	0.5	0.1111	0.4595	0.125
Glass wool	0.0034	0.3547	0.0968	0.0607	0.4167	1	0.1111	0.425	0.2727

**Table 8:** Weighted normalised matrix.

Insulation Type	VDRF	SAC	EC	EE	C	RF	SHC	TC	D
Cotton stalk fibres	0.00000805	0.0099	0.0267	0.0022	0.0623	0.004	0.0098	0.0064	0.0009
Cotton waste	0.00001012	0.0191	0.0531	0.0037	0.0607	0.004	0.0008	0.0109	0.0089
Hemp	0.00003726	0.0139	0.4855	0.0053	0.0681	0.004	0.0007	0.0055	0.0045
Kenaf	0.00001219	0.0154	0.0507	0.0032	0.0688	0.004	0.0013	0.0127	0.0027
Rice husk	0.00001357	0.0107	0.1133	0.073	0.2341	0.0237	0.0007	0.007	0.0019
Sheep wool	0.00003036	0.0145	0.5664	0.0184	0.0488	0.004	0.0009	0.0097	0.0189
Wood fibre	0.00002024	0.0065	0.5481	0.0049	0.0364	0.004	0.0006	0.0101	0.0018
Cellulose	0.00001587	0.0172	0.0635	0.0144	0.0476	0.0059	0.0009	0.0114	0.0052
Cork	0.00011845	0.0151	0.0829	0.0038	0.0332	0.004	0.0008	0.0111	0.0026
Flax	0.00002116	0.0166	0.0034	0.0025	0.0709	0.0059	0.0008	0.0073	0.0047
Vacuum-insulated panel	0.0023	0.0063	0.0079	0.0005	0.0089	0.0119	0.0016	0.0078	0.0015
Nano insulation materials	0.00003381	0.0126	0.309	0.0487	0.0004	0.0059	0.0013	0.0165	0.0012
Aerogel	0.0000253	0.016	0.0194	0.0139	0.0085	0.0119	0.0128	0.0262	0.0026
Fibreglass	0.00000782	0.0284	0.0548	0.0044	0.0975	0.0237	0.0014	0.0111	0.0052
Phenolic foam	0.00023667	0.0105	0.012	0.0012	0.0509	0.0059	0.0009	0.0212	0.0028
Polyisocyanurate	0.00069667	0.0021	0.0123	0.0014	0.0532	0.0079	0.0009	0.0194	0.0075
Extruded polystyrene	0.00084548	0.0111	0.009	0.0011	0.0571	0.004	0.0008	0.0127	0.0079
Expanded polystyrene	0.00040595	0.0427	0.01	0.001	0.0914	0.004	0.001	0.0127	0.0083
Rock wool	0.00000782	0.0145	0.0647	0.0059	0.0732	0.0119	0.0014	0.012	0.0024
Glass wool	0.00000782	0.0151	0.0548	0.0044	0.0975	0.0237	0.0014	0.0111	0.0052

**Table 9:** Ideal alternatives.

Ideal Alternative/Criteria	VDRF	SAC	EC	EE	C	RF	SHC	TC	D
	$y_{1}$	$y_{2}$	$y_{3}$	$y_{4}$	$y_{5}$	$y_{6}$	$y_{7}$	$y_{8}$	$y_{9}$
Y	0.0023	0.0427	0.5664	0.073	0.2341	0.0237	0.0128	0.0262	0.0189

**Table 10:** Decomposition of the ideal alternatives.

Ideal Alternative/Criteria	VDRF	SAC	EC	EE	C	RF	SHC	TC	D
	Max	Max	Min	Min	Min	Min	Min	Min	Min
	$y_{1}$	$y_{2}$	$y_{3}$	$y_{4}$	$y_{5}$	$y_{6}$	$y_{7}$	$y_{8}$	$y_{9}$
$Y^{max}$	0.0023	0.0427	-	-	-	-	-	-	-
$Y^{min}$	-	-	0.5664	0.073	0.2341	0.0237	0.0128	0.0262	0.0189

**Table 11:** Magnitude levels of ideal alternatives.

	Magnitude
$Y_{k}$	0.0428
$Y_{h}$	0. 6186

**Table 12:** The results of the MCRAT method.

Insulation Type	$E_{k}$	$E_{h}$	$z_{11;i}$	$z_{22;i}$	$tr\left(Z\right)$	Ranking
Cotton stalk fibres	0.0099	0.0689	0.0004	0.0426	0.043	15
Cotton waste	0.0191	0.0821	0.0008	0.0508	0.0516	12
Hemp	0.0139	0.4903	0.0006	0.3033	0.3039	3
Kenaf	0.0154	0.0866	0.0007	0.0536	0.0543	11
Rice husk	0.0107	0.2713	0.0005	0.1678	0.1683	5
Sheep wool	0.0145	0.5692	0.0006	0.3521	0.3527	1
Wood fibre	0.0065	0.5494	0.0003	0.3399	0.3402	2
Cellulose	0.0172	0.0818	0.0007	0.0506	0.0513	13
Cork	0.0151	0.0902	0.0006	0.0558	0.0564	10
Flax	0.0166	0.0718	0.0007	0.0444	0.0451	14
Vacuum-insulated panel	0.0067	0.0187	0.0003	0.0116	0.0119	20
Nano insulation materials	0.0126	0.3133	0.0005	0.1938	0.1943	4
Aerogel	0.016	0.0405	0.0007	0.0251	0.0258	19
Fibreglass	0.0284	0.1151	0.0012	0.0712	0.0724	6
Phenolic foam	0.0105	0.0568	0.0004	0.0351	0.0355	18
Polyisocyanurate	0.0022	0.059	0.0001	0.0365	0.0366	17
Extruded polystyrene	0.0111	0.0599	0.0005	0.0371	0.0376	16
Expanded polystyrene	0.0427	0.0933	0.0018	0.0577	0.0595	9
Rock wool	0.0145	0.0994	0.0006	0.0615	0.0621	8
Glass wool	0.0151	0.1151	0.0006	0.0712	0.0718	7

**Table 13:** Comparative analysis.

Insulation Type	MCRAT	MARCOS	ARAS	CRADIS
Cotton stalk fibres	15	14	14	14
Cotton waste	12	11	10	11
Hemp	3	3	3	3
Kenaf	11	12	12	12
Rice husk	5	4	4	4
Sheep wool	1	1	1	1
Wood fibre	2	2	2	2
Cellulose	13	10	9	10
Cork	10	13	11	13
Flax	14	15	16	15
Vacuum-insulated panel	20	20	20	20
Nano insulation materials	4	5	5	5
Aerogel	19	16	15	16
Fibreglass	6	6	6	6
Phenolic foam	18	17	19	17
Polyisocyanurate	17	18	18	18
Extruded polystyrene	16	18	17	19
Expanded polystyrene	9	9	13	9
Rock wool	8	8	8	8
Glass wool	7	7	7	7

## Data Availability

The data presented in this study are available upon request from the corresponding author. The data are not publicly available due to the complexity of the calculations.
